# Specificity protein 1 is a novel target of 2, 4-bis (p-hydroxyphenyl)-2-butenal for the suppression of human oral squamous cell carcinoma cell growth

**DOI:** 10.1186/1423-0127-21-4

**Published:** 2014-01-15

**Authors:** Jung-Il Chae, RaHam Lee, JinHyoung Cho, JinTae Hong, Jung-Hyun Shim

**Affiliations:** 1 Department of Dental Pharmacology, School of Dentistry, BK21 plus, Chonbuk National University, Jeonju 561-756, Republic of Korea; 2 College of Pharmacy and Medical Research Center, Chungbuk National University, 48 Gaeshin-dong, Heungduk-gu, Cheongju, Chungbuk 361-763, South Korea; 3 Department of Pharmacy, College of Pharmacy, Mokpo National University, 1666 Youngsan-ro, Muan-gun, Jeonnam 534-729, Republic of Korea

**Keywords:** 2,4-bis (p-hydroxyphenyl)-2-butenal, Apoptosis, Specificity protein 1, Oral squamous cell carcinoma, Oncology

## Abstract

**Background:**

The Maillard reaction is a chemical reaction occurring between a reducing sugar and an amino acid, generally requiring thermal processing. Maillard reaction products (MRPs) have antioxidant, antimutagenic, and antibacterial effects though 2,4-bis (p-hydroxyphenyl)-2-butenal (HPB242), a fructose-tyrosine MRP, appears to inhibit proliferation of cancer cells, its mechanism of action has not been studied in detail. The purpose of this study was to investigate the anti-proliferative effects of 2,4-bis (p-hydroxyphenyl)-2-butenal (HPB242) on two oral squamous cell carcinoma (OSCC) cell lines, HN22 and HSC4, through regulation of specificity protein 1 (Sp1).

**Results:**

HPB242 treatment dramatically reduced the cell growth rate and apoptotic cell morphologies. Sp1 was significantly inhibited by HPB242 in a dose-dependent manner. Furthermore, cell cycle regulating proteins and anti-apoptotic proteins, which are known as Sp1 target genes, were altered at the molecular levels. The key important regulators in the cell cycle such as p27 were increased, whereas cell proliferation- and survival-related proteins such as cyclin D1, myeloid leukemia sequence 1 (Mcl-1) and survivin were significantly decreased by HPB242 or suppressed Sp1 levels, however pro-apoptotic proteins caspase3 and PARP were cleaved in HN22 and HSC4.

**Conclusions:**

HPB242 may be useful as a chemotherapeutic agent for OSCC for the purpose of treatment and prevention of oral cancer and for the improvement of clinical outcomes.

## Background

Oral squamous cell carcinoma (OSCC) is a common type of malignant tumor. New cases of oral cancer occur at around 275,000 patients, and OSCCs cases comprise approximately >90% of diagnosed patients with oral cancer [1]. Although conservative treatments of oral cancer, including surgery, radiation and chemotherapy, have well advanced to date, the five-year survival rate still remains to be less than 50% [2]. Oral cancer is a serious health problem in many parts of the world and is the eighth-leading cause of cancer-related death in men. Certain studies have proposed that some of the risk factors for oral cancer are tobacco, alcohol, ultraviolet light and oral lesions [3]. Although the occurrence of oral cancer is low, the development of more effective therapeutic strategies for the prevention and treatment of oral cancer is imperative. Several studies have reported that novel plant-derived compounds act as antitumor agents through modulation of biological pathways [4].

Maillard Reaction Products (MRPs) such as Glucose-tyrosine (Glu-Tyr) and Xylose-arginine (Xyl-Arg) have antioxidant, antimutagenic, and antibacterial effects [5,6] and the MR is one of the most common and complex reactions that takes place mainly in foods during thermal processing [7]. Also many studies have reported beneficial effects associated with maillard reaction products, including antioxidative [8-10] antimicrobial, antihypertensive, anticarcinogenic, and antimutagenic properties [10-12]. However, to date, little is known about other biological effects of MRPs. In this study, we examined whether the fructose-tyrosine MRP, HPB242, could modulate cell cycle progression and Specificity protein (Sp) repression, and thus induce apoptotic cell death of OSCCs.

Sp is a transcription factor and universally expressed in all mammalian cells [13]. Specificity protein 1 (Sp1) was recently defined as the Sp/krűppel-like transcription factor [14] and was identified to play a significant role in various physiological processes such as cell cycle regulation, apoptosis and angiogenesis [15-18]. Furthermore, Sp1 is highly expressed in various cancers such as breast carcinoma, thyroid cancer, hepatocellular carcinoma, pancreatic cancer, colorectal cancer, gastric cancer, cervical cancer and lung cancer [16,18-21]. Regarding its cancer-related functions, Sp1 has been suggested to be a novel target for cancer therapy.

To characterize the effect of 2,4-bis (p-hydroxyphenyl)-2-butenal (HPB242) on OSCCs, this study specifically examined the anti-cancer effect of HPB242 on cell viability against two oral squamous cell carcinoma cell lines, HN22 and HSC4, and identified regulated proteins by HPB242 treatment in the cells.

In this study, we investigated whether downstream proteins of Sp1 protein and key apoptotic proteins could be affected in their expression toward apoptotic cell death through alteration of Sp1 expression by HPB242 treatment. Our results provide evidence for the chemotherapeutic efficacy of HPB242 in oral squamous cells.

## Methods

### Cell culture

HN22 and HSC4 cells, a type of human oral squamous cancer cells, were obtained from Dankook University (Cheonan) and Hokkaido University (Hokkaido) respectively. HN22 and HSC4 were cultured in Hyclone Dulbecco’s modified Eagle’s medium (DMEM) containing 10% heat-inactivated fetal bovine serum and 100U/ml each of penicillin and streptomycin at 37°C with 5% CO_2_ in humidified air.

### Cell viability assay

Cell viability of HN22 and HSC4 cells was accessed using the trypan blue dye exclusion method [22,23]. Briefly, both HN22 and HSC4 cells were seeded on a 6-well microtiter plate (5 × 10^4^ cells/well), after which they were treated with different doses of 5, 10, 15 and 20 μg/ml HPB242 for 24 hours and 48 hours. The HN22 and HSC4 cells treated with HPB242 were harvested by trypsinization and washed in cultured media. Trypan blue (0.4%) was added to treated cells, and after 5 min, cells were loaded into a hemocytometer and counted. The number of viable cells was calculated as percent of the total cell population.

### DAPI staining

The number of undergoing apoptotic cells by HPB242 was quantified using 4′-6-diamidino-2-phenylindole (DAPI) staining. Nuclear condensation and fragmentation were determined by DAPI stained nucleic acid. After 48 hours post-treatment of the agent with different doses (HPB242; 5, 10, and 20 μg/ml), HN22 and HSC4 cells were harvested by trypsinization, washed with cold phosphate buffered saline (PBS), and fixed in 100% methanol at room temperature for 20 minutes. The cells were spread on a slide and then stained with DAPI (2 μg/ml), and subsequently monitored by a FluoView confocal laser microscope (Fluoview FV10i, Olympus Corporation, Tokyo).

### Propidium iodide staining

After 48 hours of HPB242 treatment in HN22 and HSC4 cells, the cells were washed with cold PBS, pooled and centrifuged before being fixed in 70% ice-cold ethanol overnight at -20°C, and then treated with 150 μg/ml RNase A and 20 μg/ml propidium iodide (PI; Sigma-Aldrich, Inc. St. Louis, Missouri). The stained cells were analyzed, and the distribution of the cells in different phases of the cell cycle was calculated using flow cytometry with a MACSQuant Analyzer (Miltenyi Biotec GmbH, Bergisch Gladbach).

### Reverse transcription-polymerase chain reaction

Total RNA was extracted from the cells using TRIzol® Reagent (Life Technologies, Carlsbad, California), and 2 μg of RNA was used to synthesize cDNA using the HelixCript™ 1st-strand cDNA synthesis kit (NanoHelix, Korea). cDNA was obtained by PCR using β-actin-specific and Sp1-specific primers as described below under following PCR conditions (35 cycles: 1 min at 95°C, 1 min at 56°C, and 1 min at 72°C). The β-actin primers used were; forward 5′ GTG GGG CGC CCC AGG CAC CA 3′ and reverse 5′ CTC CTT AAT GTC ACG CAC GAT TTC 3′; and the Sp1 primers were; forward 5′ ATG CCT AAT ATT CAG TAT CAA GTA 3′ and reverse 5′ CCC TGA GGT GAC AGG CTG TGA 3′. PCR products were analyzed by 1% agarose gel electrophoresis.

### Western blot analysis

Total cell lysate was prepared using PRO-PREP™ Protein Extraction Solution (iNtRON Biotechnology, Korea) containing 1 μg/ml aprotinin, 1 μg/ml leupeptin, and 1 mM PMSF. Fifty micrograms of total protein was separated via 10 or 15% (v/v) SDS-polyacrylamide gel electrophoresis and then transferred onto polyvinylidene difluoride (PVDF) membranes. After blocking for two hours at room temperature with 5% non-fat dried milk in PBST containing 0.1% tween-20, the membranes were immunoblotted with specific primary antibodies against Sp1 (1C6), p27 (C-19), Cyclin D1 (M-20) (Santa Cruz Biotechnology, Santa Cruz, CA), PARP (BD Biosciences, San Diego, CA), Mcl-1, Survivin, Cleaved-caspase3, anti-Bid, anti-Bax, anti-Bcl-_xl_ (Cell Signaling, Danvers, MA) and β-actin (AC-74) (Sigma-Aldrich) overnight at 4°C. After washing with PBST, secondary antibodies to IgG (Santa Cruz Biotechnology) conjugated with horseradish peroxidase were used, and chemiluminescence signals were enhanced with the Pierce ECL Western Blotting Substrate (Thermo scientific, Rockford) according to the manufacturer’s instructions.

### Immunocytochemistry analysis

The cells were seeded over each sterilized glass coverslips on six-well tissue culture plates for 24 hours and incubated with HPB242 for 48 hours. The cells were fixed and permeabilized with Cytofix/cytoperm solution for 30 min. For Sp1 and Cleaved-caspase3 expression, the cells were blocked with 1% BSA and then incubated with monoclonal Sp1 and Cleaved-caspase3 antibody at 4°C overnight. After washing with PBST solution, the Sp1 and Cleaved-caspase3 antibody was reacted with a Jackson 488- and 647-conjugated anti-mouse secondary antibody at room temperature for 1 h and then mounted with Mountin solution-VECTSHIELD mounting medium for fluorescence with DAPI (Vector Laboratories, Inc. Burlingame, CA) onto the cells. The cells were visualized using a FluoView confocal laser microscope.

### Statistical analysis

Data are reported as the mean ± SD of at least three independent experiments. Statistical significance was evaluated using Student’s t-test. Compared to the vehicle control, *p* < 0.05 were considered significant.

## Results

### Growth inhibition effects of HPB242 on OSCCs

**Figure 1:**
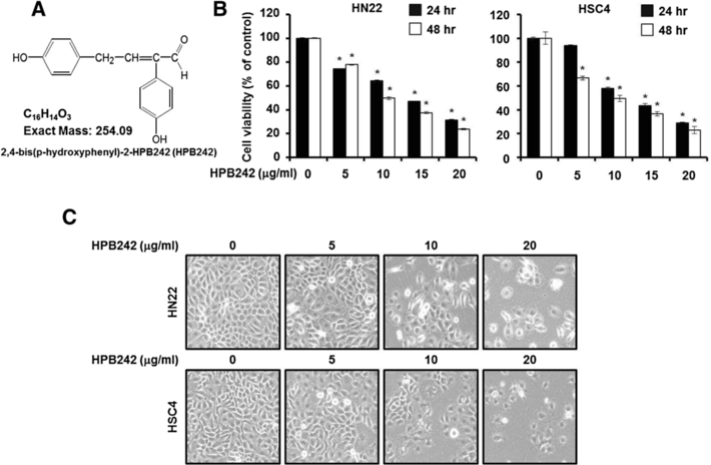
**The effect of HPB242 on cell viability of oral cancer cells. (A)** Chemical structure of HPB242. **(B)** The cell viability effect of HPB242 on HN22 and HSC4 cells. HN22 cells (2 × 10^3^ cells/well) and HSC4 (3 × 10^3^ cells/well) cells were seeded in 96-well plates and incubated for 24 hours and 48 hours with increasing concentrations of HPB242 in 10% FBS-DMEM. Cell viability was estimated using the trypan blue dye exclusion method, as described under Methods. Results are indicated as cell viability relative to the untreated HPB242, and data are represented as means ± SD from three independent experiments. The asterisk indicates a significant difference compared to the negative control (untreated cells) (*p < 0.05). **(C)** Morphological changes observed in the HPB242 treated (5, 10, and 20 μg/ml) or untreated HN22 and HSC4 cells 48 hours post-treatment.

To determine the effect of HPB242 on the cell viability of two OSCCs, HN22 and HSC4, we confirmed the growth inhibitory potential by trypan blue assay. The results indicated that HPB242 decreased the viability of HN22 and HSC4 cells in a dose-and time-dependent for 24 and 48 hours (Figure 1B). The IC_50_ value of HPB242 for 48 hours of incubation was estimated as 9.96 μg/ml in HN22 and 9.85 μg/ml in HSC4 cells, respectively. The cell viabilities of HN22 were shown as 78 ± 0.3%, 49.8 ± 1.2%, 37.5 ± 0.8%, and 23.8 ± 0.5% at 5, 10, 15, and 20 μg/ml of HPB242, respectively, compared with untreated control cells when viabilities were calculated at 48 hours post-treatment. In the case of HSC4, viabilities were measured as 66.7 ± 0.9%, 49.5 ± 0.5, 36.7 ± 0.6%, and 22.9 ± 1.0% at 5, 10, 15, and 20 μg/ml of HPB242, respectively, compared to that of untreated control cells when viabilities were calculated at 48 hours post-treatment. Apoptosis-induced changes in the cell morphology in the HPB242-containing medium were observed. After 48 hours, the apoptotic phenotype showed cell rounding, cytoplasmic blebbing and irregularities in shape, indicating a sharp increase in the apoptosis of HPB242-treated HN22 and HSC4 cells in a concentration-dependent manner (Figure 1C).

### HPB242 treatments induces apoptosis of OSCCs

**Figure 2:**
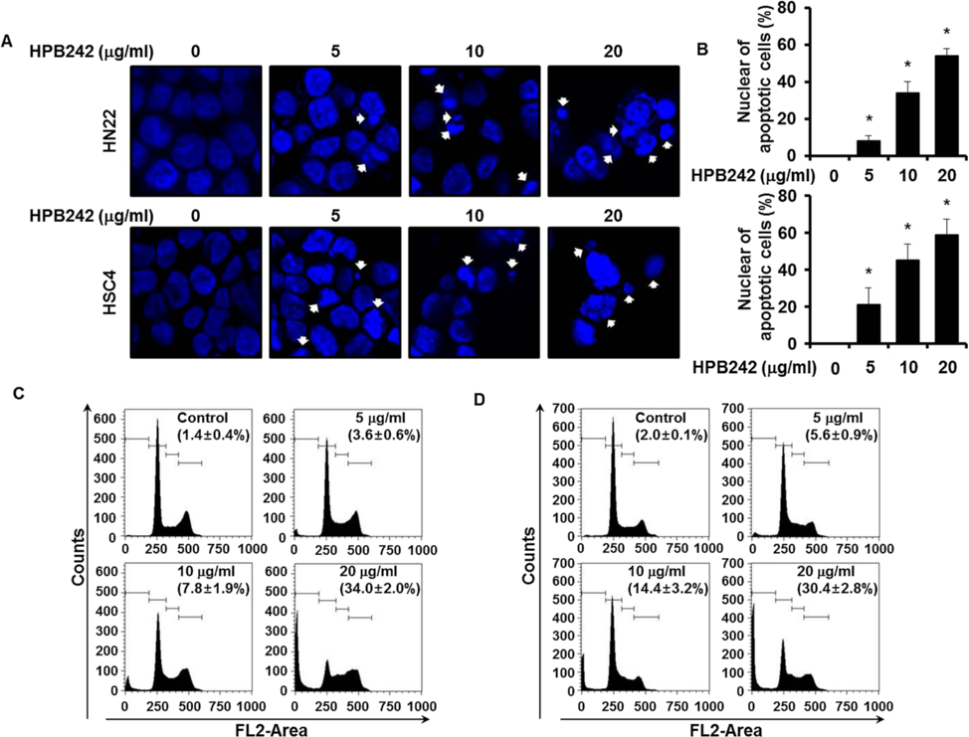
**The apoptotic effect induced by HPB242 in HN22 and HSC4 cells.** Cells were incubated with HPB242 (5, 10, and 20 μg/ml) and untreated for 48 hours. The cells were harvested and prepared for DAPI staining and PI staining as described in the Methods section. **(A)** Analysis of DNA fragmentation and nuclear condensation by fluorescence microscopy (magnification ×600) after HPB242 treatment in HN22 and HSC4 cells. **(B)** DNA fragmentation and nuclear condensation were quantified, and the results in triplicate are expressed as the mean ± SD. **(C and D)** Representative histograms of Sub-G1 population. HPB242-treated cells were compared with untreated cells, and data are shown as the average of triplicate samples from three independent experiments. The asterisks (*) indicates *p* < 0.05 versus control cells.

The effect of HPB242 treatment on initiation was due to the induction of apoptotic cell death by nuclear morphology using DAPI staining, which allowed visualization of nuclear shrinkage and fragmentation. HPB242 treatment of HN22 and HSC4 cells led to an increase in nuclear condensation and fragmentation compared to the control group (Figure 2A, B). In order to determine whether Sub-G1 population induction by HPB242 is related to apoptosis, HPB242 treated cells were marked with PI staining. When HN22 and HSC4 cells were treated with HPB242, an increased number of cells in the Sub-G1 population was observed in 3.6 - 34% of HPB242-treated HN22 cells and in 5.6 – 30.4% of HPB242-treated HSC4 cells (Figure 2C, D).

### Specificity protein 1 protein is suppress by HPB242

**Figure 3:**
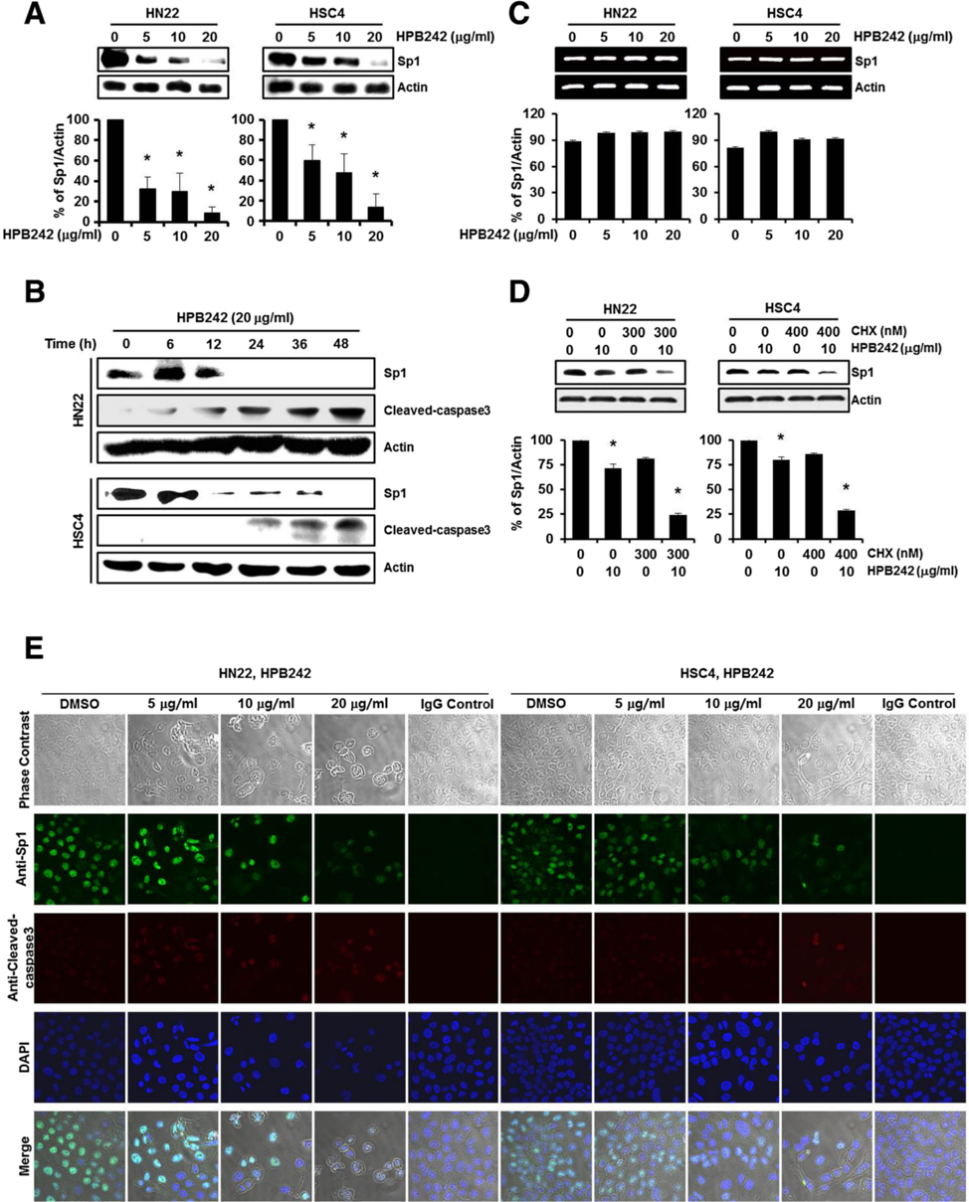
**The effect of HPB242 on specificity protein 1 (Sp1) protein expression in HN22 and HSC4 cells.** HN22 and HSC4 cells were incubated with different concentrations of HPB242 for 48 hours. The cells were harvested and prepared for western blots as described under Methods. Protein expression levels of Sp1 were detected using a specific antibody against Sp1, and its levels were quantified after actin normalization. **(A)** The HPB242-treated cells were compared with untreated cells, and data are shown as the means ± SD of three independent experiments. The asterisk indicates a significant difference compared with the negative control (untreated cells) (*p < 0.05). **(B)** Time-dependent effects of HPB242 on Sp1 and Cleaved-caspase3 expression were performed in HN22 and HSC4 cells for 48 hours with 6 hours intervals. **(C)** The effect of HPB242 (0–20 μg/ml) for 48 hours on Sp1 mRNA expression was determined by RT-PCR. The graphs indicate the ratio of Sp1 to β-actin expression. **(D)** The effect of HPB242 on Sp1 protein turnover in HN22 and HSC4 cells. The protein lysates were obtained from cells pretreated with protein synthesis inhibitor such as cycloheximide (CHX) for 2 hour and then exposed to HPB242 for 48 hours. The protein expression of Sp1 was analyzed by western blot analysis. **(E)** Immunocytochemistry analysis was performed in HPB242 treated HN22 and HSC4 cells. HN22 and HSC4 cells were treated with different concentrations of HPB242 for 48 hours, and cells were immunostained with Sp1 specific antibody, Cleaved-caspase3 specific antibody, and then signals were detected with Jackson 488- and 647-conjugated anti-mouse secondary antibody. DAPI was used for nucleus staining. 254×190 mm (96 × 96 DPI).

**Figure 4:**
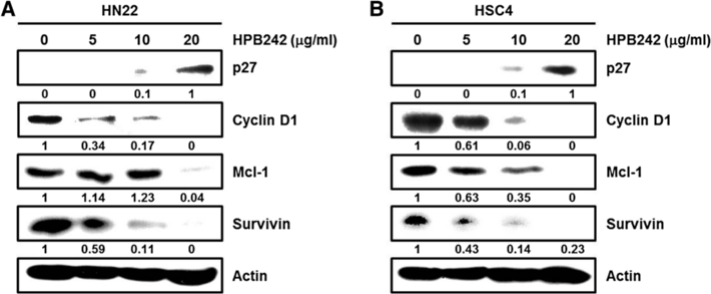
**The effect of HPB242 on downstream target proteins by the specificity protein 1 (Sp1).** HN22 **(A)** and HSC4 **(B)** cells were incubated with HPB242 (0, 5, 10 and 20 μg/ml) for 48 h. The effect of HPB242 for 48 hours on p27, Cyclin D1, Mcl-1, and Survivin was determined by Western blots. Fifty micrograms of cellular extract per lane was separated on an SDS-PAGE gel as described in Methods. Equal loading of protein was confirmed by incubating the same membrane with anti-β-Actin antibody.

Several studies have recognized that the expression levels of transcription factor Sp1 dramatically increases during transformation, representing a critical effect in tumor development or maintenance. The effects of HPB242 treatment on Sp1 levels were inspected by western blotting. As shown in Figure 3A, HPB242 treatment led to a sharp decrease in the level of Sp1 in HN22 and HSC4 cells at 48 hours after treatment. In order to characterize the apoptotic action of HPB242, we confirmed expression levels of Cleaved-caspase3 by western blotting (Figure 3B). However, Sp1 mRNA levels did not suppressed by HPB242 in both HN22 and HSC4 cells (Figure 3C). When CHX-pretreated HN22 and HSC4 cells were incubated with HPB242, degradation of Sp1 protein by HPB242 was additionally enhanced (Figure 3D). There were increases in Cleaved-caspase3 following HPB242 (20 μg/ml) treatment of HN22 and HSC4 cells in a time-dependent manner. According to these observations, immunocytochemical results also showed decreased levels of Sp1 positive cells in a dose-dependent manner in HN22 and HSC4 cells (Figure 3E). HPB242 also cell cycle arrest-related proteins such as p27 were increased, whereas cell proliferation- and survival-related proteins such as cyclin D1, Mcl-1 and survivin were significantly decreased (Figure 4A, B). These results collectively suggest that down-regulation of Sp1 by HPB242 treatment could lead to apoptotic cell death.

### HPB242 regulate the expression of anti-apoptotic and apoptotic molecules in OSCCs

**Figure 5:**
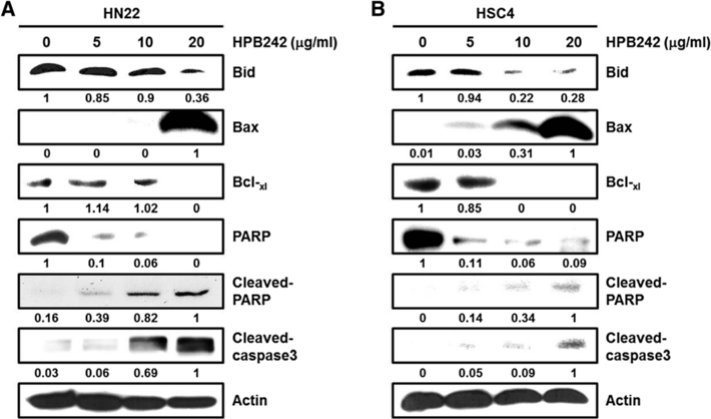
**The effect of HPB242 on apoptosis of HN22 and HSC4 cells.** HN22 **(A)** and HSC4 **(B)** cells were incubated with HPB242 (0, 5, 10 and 20 μg/ml) for 48 h. Fifty micrograms of cellular extract per lane was separated on an SDS-PAGE gel as described in Methods. Immunoblot detection of Bid, Cleaved-caspase3, and PARP, upregulation of Bax, downregulation of Bcl-_xl_ in whole cell lysates from various concentration HPB242-treated HN22 and HSC4 cells for 48 hours. Equal loading of protein was confirmed by incubating the same membrane with anti-β-Actin antibody.

Treatment of cells with HPB242 regulates the expression levels of various apoptotic proteins (Figure 5A, B). An immunoblotting technique was used to analyze the levels of several pro- and anti-apoptotic proteins to determine whether treatment with HPB242 regulates the expression of apoptosis-related proteins in HN22 and HSC4 cells. Activated Bid, PARP, and Bcl-_xl_ were decreased and Bax, Cleaved-PARP and Cleaved-caspase3 increased in HPB242-treated HN22 and HSC4 cells.

## Discussion

MRPs such as Glu-Tyr and Xyl-Arg have antioxidant, antimutagenic, and antibacterial effects [24]. Although HPB242, a fructose-tyrosine MRP, appears to inhibit proliferation of cancer cells, its mechanism of action has not been studied in detail.

In this study, our findings along with the findings of previous research, HPB242 may induce apoptosis in OSCCs. In the present study, the apoptotic effects of HPB242 on HN22 and HSC4 cells were investigated through the trypan blue dye exclusion method. This assay, a traditional method for discovering anticancer drugs, can be used to determine the cytotoxic effect and proliferation of cell lines [25,26]. In this study, this assay was used to elucidate the apoptotic effects of HPB242 on HN22 and HSC4 cells (Figure 1). As shown in Figure 2, HPB242 inhibited the proliferation of HN22 and HSC4 cells through cell cycle arrest at G0/G1 and induction of apoptosis.

Transcription factor Sp1 is known to be regulated by molecular target genes in various biological processes including differentiation, metabolism, cell growth, angiogenesis and apoptosis [13]. Therefore, Sp1 protein levels are expected to be a negative prognostic factor and a potential therapeutic target for cancer chemotherapy [27].

As shown in Figure 3A, B, treatment with HPB242 induced a significant decrease in the protein expression levels of Sp1 in the HN22 and HSC4 cells in a dose- and time-dependent manner. However, Sp1 mRNA did not suppressed by HPB242 in both HN22 and HSC4 cells (Figure 3C). When CHX-pretreated HN22 and HSC4 cells were incubated with HPB242, degradation of Sp1 protein by HPB242 was additionally enhanced (Figure 3D). Immunocytochemistry results also revealed a decreased level of Sp1 and an increased level of Cleaved-caspase3 in a dose-dependent manner in the HN22 and HSC4 cell lines (Figure 3E).

Our results confirmed that HPB242 induced nuclear condensation and apoptosis of HN22 and HSC4 cells and inhibit the expression of Sp1 and Sp1 regulatory proteins. In a previous study, HPB242 inhibited cell viability and induced apoptotic cell death in HN22 and HSC4 cells [13]. In addition, HPB242 inhibited the transcriptional activity and expression of Sp1 downstream proteins, including p27, Cyclin D1, Mcl-1 and Survivin, in a dose-dependent manner (Figure 4). HPB242 reduced Bid and Bcl-_xl_, increased Bax, and activated Caspase3 and PARP, suggesting that HPB242 regulates Sp1 and ultimately leads to apoptotic cell death (Figure 5). Our results demonstrate Sp1 could serve as an efficient therapeutic target of cancer. Sp1 expression levels increase during transformation, which can play a critical role in tumor development or maintenance. Although Sp1 deregulation is beneficial for treating tumor cells, it is reported that overexpression of Sp1 induces apoptosis of untransformed cells or cancer cells [17].

To further confirm whether HPB242 could modulate anti-apoptotic protein expression toward apoptosis, we observed alterations of Mcl-1 and Survivin when cells were treated with different doses. Mcl-1, Survivin and cell cycle regulatory proteins were greatly reduced by HPB242 treatment in a dose dependent manner (Figure 4). Thus, HPB242 can be said to positively regulate p27 and negatively regulate Cyclin D1, Mcl-1 and Survivin in OSCCs, resulting in activation of a caspase-dependent apoptosis pathway through Cleaved-caspase3 and PARP (Figure 5). In this study, we investigated the cancer chemoprevention effect of HPB242 on OSCCs. Our results revealed that HPB242 has cell growth inhibitory activity and induces apoptosis in OSCCs through inhibition of Sp1 expression.

Taken together, clinical studies with HPB242 are necessary to explain its unexpected potential toxicity and clinical applications.

## Conclusion

OSCC was influenced by the chemotherapeutic effects of HPB242. We suggest that HPB242 regulate Sp1 target proteins, resulting in apoptosis by the suppression of Sp1 levels in HN22 and HSC4 cells. Sp1 can be used as an effective therapeutic target in cancer research, and HPB242 are potential cancer drugs or adjuvants as chemotherapeutic agents for OSCC.

## Competing interests

The authors declare that they have no competing interests.

## Authors’ contributions

J-HS designed research and wrote the manuscript. J-IC, RHL and JHJ carried out research experiments and analyzed data. JTH and J-IC participated in the design and performance of this study. All authors read and approved the final manuscript.

## References

[B1] Hamada T Wakamatsu T Miyahara M Nagata S Nomura M Kamikawa Y Yamada N Batra SK Yonezawa S Sugihara K MUC4: a novel prognostic factor of oral squamous cell carcinoma Int J Cancer J Int du cancer 2012 130 1768 1776 10.1002/ijc.26187 21618516

[B2] Warnakulasuriya S Global epidemiology of oral and oropharyngeal cancer Oral Oncol 2009 45 309 316 10.1016/j.oraloncology.2008.06.002 18804401

[B3] Mashberg A Boffetta P Winkelman R Garfinkel L Tobacco smoking, alcohol drinking, and cancer of the oral cavity and oropharynx among U.S. veterans Cancer 1993 72 1369 1375 10.1002/1097-0142(19930815)72:4<1369::AID-CNCR2820720436>3.0.CO;2-L 8339227

[B4] Gupta SC Kim JH Prasad S Aggarwal BB Regulation of survival, proliferation, invasion, angiogenesis, and metastasis of tumor cells through modulation of inflammatory pathways by nutraceuticals Cancer Metast Rev 2010 29 405 434 10.1007/s10555-010-9235-2 PMC2996866 20737283

[B5] Wang WQ Bao YH Chen Y Characteristics and antioxidant activity of water-soluble Maillard reaction products from interactions in a whey protein isolate and sugars system Food Chem 2013 139 355 361 10.1016/j.foodchem.2013.01.072 23561117

[B6] Hwang IG Kim HY Woo KS Hong JT Hwang BY Jung JK Lee J Jeong HS Isolation and characterisation of an alpha-glucosidase inhibitory substance from fructose-tyrosine Mail lard reaction products Food Chem 2011 127 122 126 10.1016/j.foodchem.2010.12.099

[B7] Yilmaz Y Toledo R Antioxidant activity of water-soluble Maillard reaction products Food Chem 2005 93 273 278 10.1016/j.foodchem.2004.09.043

[B8] Lertittikul W Benjakul S Tanaka M Characteristics and antioxidative activity of Maillard reaction products from a porcine plasma protein-glucose model system as influenced by pH Food Chem 2007 100 669 677 10.1016/j.foodchem.2005.09.085

[B9] Maillard MN Billaud C Chow YN Ordonaud C Nicolas J Free radical scavenging, inhibition of polyphenoloxidase activity and copper chelating properties of model Maillard systems Lwt-Food Sci Technol 2007 40 1434 1444 10.1016/j.lwt.2006.09.007

[B10] Rufian-Henares JA Morales FJ Angiotensin-I converting enzyme inhibitory activity of coffee melanoidins J Agricult Food Chem 2007 55 1480 1485 10.1021/jf062604d 17243703

[B11] Manzocco L Calligaris S Mastrocola D Nicoli MC Lerici CR Review of non-enzymatic browning and antioxidant capacity in processed foods Trends Food Sci Tech 2000 11 340 346 10.1016/S0924-2244(01)00014-0

[B12] Yen GC Tsai LC Antimutagenicity of a partially fractionated maillard reaction-product Food Chem 1993 47 11 15 10.1016/0308-8146(93)90295-Q

[B13] Li L Davie JR The role of Sp1 and Sp3 in normal and cancer cell biology Ann Anatomy 2010 192 275 283 10.1016/j.aanat.2010.07.010 20810260

[B14] Wan J Carr BA Cutler NS Lanza DL Hines RN Yost GS Sp1 and Sp3 regulate basal transcription of the human CYP2F1 gene Drug Metab Dispos: Biol Fate Chem 2005 33 1244 1253 10.1124/dmd.105.004069 15860659

[B15] Chu S Ferro TJ Sp1: regulation of gene expression by phosphorylation Gene 2005 348 1 11 15777659 10.1016/j.gene.2005.01.013

[B16] Chuang JY Wu CH Lai MD Chang WC Hung JJ Overexpression of Sp1 leads to p53-dependent apoptosis in cancer cells Int J Cancer J Int du Cancer 2009 125 2066 2076 10.1002/ijc.24563 19588484

[B17] Deniaud E Baguet J Mathieu AL Pages G Marvel J Leverrier Y Overexpression of Sp1 transcription factor induces apoptosis Oncogene 2006 25 7096 7105 10.1038/sj.onc.1209696 16715126

[B18] Chen L Liu Q Qin R Le H Xia R Li W Kumar M Amplification and functional characterization of MUC1 promoter and gene-virotherapy via a targeting adenoviral vector expressing hSSTR2 gene in MUC1-positive Panc-1 pancreatic cancer cells in vitro Int J Mol Med 2005 15 617 626 15754023

[B19] Davie JR He S Li L Sekhavat A Espino P Drobic B Dunn KL Sun JM Chen HY Yu J Nuclear organization and chromatin dynamics–Sp1, Sp3 and histone deacetylases Adv Enzyme Regulat 2008 48 189 208 10.1016/j.advenzreg.2007.11.016 18187045

[B20] Kong LM Liao CG Fei F Guo X Xing JL Chen ZN Transcription factor Sp1 regulates expression of cancer-associated molecule CD147 in human lung cancer Cancer Sci 2010 101 1463 1470 10.1111/j.1349-7006.2010.01554.x 20384626 PMC11159187

[B21] Sankpal UT Goodison S Abdelrahim M Basha R Targeting Sp1 transcription factors in prostate cancer therapy Med Chem 2011 7 518 525 10.2174/157340611796799203 22022994

[B22] Liu P Wang X Hu C Hu T Inhibition of proliferation and induction of apoptosis by trimethoxyl stilbene (TMS) in a lung cancer cell line Asian Pacif J Cancer Prev: APJCP 2011 12 2263 2269 22296367

[B23] Kang NJ Lee KW Rogozin EA Cho YY Heo YS Bode AM Lee HJ Dong Z Equol, a metabolite of the soybean isoflavone daidzein, inhibits neoplastic cell transformation by targeting the MEK/ERK/p90RSK/activator protein-1 pathway J Biolog Chem 2007 282 32856 32866 10.1074/jbc.M701459200 17724030

[B24] Kim MS Kim JH Bak Y Park YS Lee DH Kang JW Shim JH Jeong HS Hong JT Yoon do Y 2,4-bis (p-hydroxyphenyl)-2-butenal (HPB242) induces apoptosis via modulating E7 expression and inhibition of PI3K/Akt pathway in SiHa human cervical cancer cells Nutr Cancer 2012 64 1236 1244 10.1080/01635581.2012.718405 23163851

[B25] Riss TL Moravec RA Use of multiple assay endpoints to investigate the effects of incubation time, dose of toxin, and plating density in cell-based cytotoxicity assays Assay Drug Dev Technol 2004 2 51 62 10.1089/154065804322966315 15090210

[B26] Li Y Huang W Huang S Du J Huang C Screening of anti-cancer agent using zebrafish: comparison with the MTT assay Biochem Biophys Res Commun 2012 422 85 90 10.1016/j.bbrc.2012.04.110 22560901

[B27] Chang WC Hung JJ Functional role of post-translational modifications of Sp1 in tumorigenesis J Biomed Sci 2012 19 94 10.1186/1423-0127-19-94 23148884 PMC3503885

